# A Brief Review on Factors Affecting the Tribological Interaction between Human Skin and Different Textile Materials

**DOI:** 10.3390/ma15062184

**Published:** 2022-03-16

**Authors:** Brian D’Souza, Ashish K. Kasar, Jaycob Jones, Andre Skeete, Lane Rader, Pankaj Kumar, Pradeep L. Menezes

**Affiliations:** 1 Department of Mechanical Engineering, University of Nevada, Reno, NV 89557, USA; bdsouza@nevada.unr.edu (B.D.); akasar@nevada.unr.edu (A.K.K.); jonesjaycob@gmail.com (J.J.); andre.p.skeete@gmail.com (A.S.); lanerader@nevada.unr.edu (L.R.); 2 Department of Chemical and Materials Engineering, University of Nevada, Reno, NV 89557, USA; pankaj@unm.edu; 3 Department of Mechanical Engineering, University of New Mexico, Albuquerque, NM 87131, USA

**Keywords:** skin, coefficient of friction, fabrics, blisters

## Abstract

The application of tribology is not just limited to mechanical components of engineering systems. As a matter of fact, the understanding of friction and wear can be applied to everyday life. One of the important fields is skin tribology, as human skin interacts with various surfaces of different materials. This paper focuses on the friction behavior of the skin when in contact with the fabric and other materials in relative motion. The excessive friction at the fabric-skin interface may lead to discomfort, blistering, chafing, and pressure ulcers especially in athletes who experience higher friction due to rapid movement for an extended period. Other than understanding the fabric properties, it is equally important to understand the structure and properties of the skin to evaluate its function and interaction with the different fabric materials. The identification of the contributing factors of skin friction can help to design suitable fabric materials. An overview of skin functions and the factors that affect the friction on the skin–textile material interface are presented in this review article.

## 1. Introduction

Human skin is a highly complex living material in that it is the largest organ within the entire body and is responsible for many functions. The primary functions of the skin are protection against foreign objections and intrusions, thermo-regulation, and retention of water. These functions are directly related to the bio-tribological properties of human skin. On a daily basis, human skin comes into contact with various materials, textures, textiles, lubricants, and atmospheric conditions. The movement of these different materials over skin causes friction. Frictional behavior of human skin changes depending on various factors, such as age, gender, weather conditions, stress levels, and the counter material/textiles [1,2,3,4]. The skin is the first line of defense on the human body, and it is exposed to friction and pressure in nearly every setting. Friction and pressure often cause skin trauma such as abrasions, chafing, calluses, and blisters. Athletes move different parts of their body more frequently due to intense activities and are therefore subjected to higher friction. Their skin can come into contact with equipment, clothing, or playing surfaces that have different textures and interact differently with each of them. Chafing and friction blisters are common challenges faced by athletes that perform high-intensity activities. The most common place for blisters to form is on the hands and feet, while in areas such as the armpit, nipples, and groin, chafing can easily occur [5]. These injuries due to textile–skin interaction may occur anywhere on the body and are not limited to the places aforementioned.

**Figure 1:**
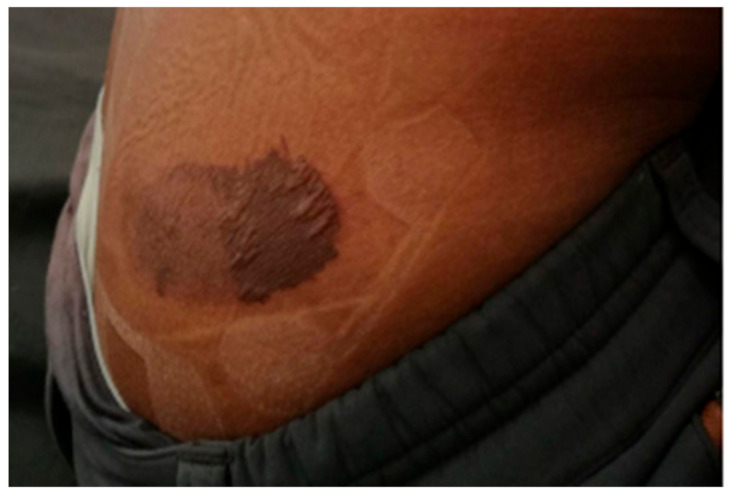
A picture of the friction blister on the 24-year-old, Mt. Everest guide, after a week of medical care [6]. Reproduced with permission from Adhikari, S.; Dawadi, S.; Upadhyay, J., Friction Blister by Climbing Harness: A Case Report; published by Elsevier, 2019.

An excellent example of friction from textile–skin interaction can be seen on an experienced mountain climber who developed blisters on his right flank, as shown in Figure 1. The blister developed at the location where the climbing harness would be to support the climber. It was speculated that a layer of clothing against his skin was bunched up underneath the harness on this particular trip, which had caused great friction on the skin and caused a large friction blister [6]. Any injury in such an extreme environment, such as Mt. Everest, can have serious consequences if left unattended. Friction blisters are a common factor among wilderness athletes and have proven to be a crippling factor in performance as they affect mobility. So, these injuries may be particularly risky if one is in a remote region where medical help is not available [7]. If textile–skin interaction can be better understood, these types of injuries may be preventable in the future, allowing for more comfort and better performance, whether it be a morning jog or a high-intensity marathon. 

According to a paper by Gerhardt et al. [2] regarding friction of human skin against textiles, “friction and shear forces, as well as moisture between the human skin and textiles, are critical factors in the formation of skin injuries such as blisters, abrasions, and decubitus”. Today, there are thousands of different textiles which make up the clothing that is worn all over the world. Even though many of the natural materials used in modern clothing were also used thousands of years ago by humans, different processing and manufacturing techniques are now used to produce long-lasting, high-performance outerwear. With advances in technology, synthetic materials have also been created in the past century which were never possible to make before. New and experimental fabrics and textiles are being researched and developed to bring them into production. Dong et al. [8] studied dual-layer nano fibrous non-woven mats that can be used to design sports textiles that provide low friction. The mat was made using polyacrylonitrile (PAN) for the core and poly(vinylidene fluoride) (PVDF) for the shell. Another advance in modern textiles is the fact that the same type of material may be processed and manufactured in a variety of ways that can provide certain desired properties to meet consumer needs. It is important to understand how skin will be affected by different materials in order to maintain optimal performance and reduce the likelihood of irritation such as chafing, blisters, and other related injuries in order to help individuals remain comfortable whether they are high performance athletes, bedridden hospital patients, or average people performing day-to-day tasks. 

This paper aims to provide an understanding of how different materials, under certain conditions, interact with human skin. The underlying goal will be to understand factors contributing to friction at the textile–skin contact interface and reducing this friction. Some of these factors include how age, humidity, skin moisture, use of different materials, interaction with various anatomical regions, and gender may play roles in influencing friction on the skin–textile material interface [1,2,3,4]. A review of how different materials perform with respect to interfacial friction and the testing methods are also presented. This allows for an analysis of how attire may need to change based on different climates and surroundings.

## 2. Human Skin

**Figure 2:**
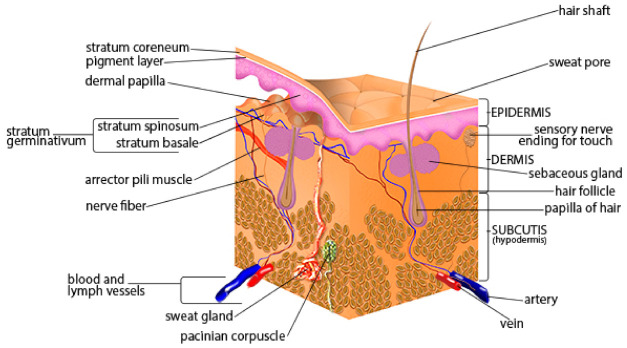
Schematic of human skin anatomy [9]. Reproduced with permission from SEER Training Modules, Skin Cancer: Melanoma; published by U.S. National Institutes of Health, National Cancer Institute.

Human skin is made up of various, highly complex layers and sublayers, each working together with their functions, as shown in Figure 2. We can separate the human skin into three primary layers, the upper epidermis, the middle dermis, and the lower hypodermis. The primary function of the epidermis is to provide a barrier from exterior conditions. The dermis houses nerve endings, glands, connective tissues, hair follicles, and blood capillaries. Additionally, the hypodermis stores fats, which provide insulation and cushion the skin. Furthermore, we can separate the main layers into various sub-layers and subsections.

The epidermis is the outermost layer of skin. It is a waterproof, protective layer that wraps over the entirety of the skin’s surface. The epidermis acts as a physical, chemical, and immunological barrier that protects against infection, external chemicals, pathogens, bacteria, and physical intrusions [10]. The epidermis can be broken down into a few different sub-layers. These include stratum corneum, stratum lucidum, stratum granulosum, stratum spinosum, and stratum basale [11]. 

The stratum corneum is where most skin contact occurs and is the epidermis’s sublayer most responsible for keeping water in the body and protecting the body from harmful pathogens and chemicals [12]. The stratum corneum is the area of skin overlaid by 25–30 layers of flattened, dead keratinocytes, a type of cell contained in the epidermis. These cells comprise about 90% of the cells within the epidermis [13]. Keratinocytes are formed when certain cells go through a process of keratinization where cells migrate from a lower sub-layer called the stratum basale. These cells replace the dead cells on the stratum corneum, and the dead cells uppermost of the stratum corneum are removed by skin shedding [14]. Furthermore, the stratum corneum is also responsible for skin hydration through the monitoring of transepidermal water loss (TWL). This water loss through the epidermal layer is affected by activity in the sweat glands, temperature, and one’s metabolism [15].

The stratum lucidum is an extra layer of skin found on the palms of hands and the soles of feet, and it is also referred to as “thick skin” [16]. Similar to the stratum corneum, this layer is also overlaid by dead, flattened keratinocytes. However, this layer contains about three to five layers of keratinocytes [13]. Deeper into the skin is the dermis, the human skin’s second main layer. The dermis lies beneath the epidermis and is composed of connective tissues and elastic fibers. The dermis is responsible for providing the epidermis nourishment and mechanical support [17]. In addition, the dermis further supports the skin by harboring and protecting the skin’s deeper layers (Figure 2). Within the dermis are two sub-layers. The uppermost layer is the papillary layer. The papillary layer is composed of loose connective tissue and cushions the body from stress and strain. Blood vessels in this layer also help thermoregulate the body and dispose of cellular waste which would otherwise kill skin cells [18]. The lower primary layer is the reticular layer. This elastic layer is the majority of the dermis and is constructed of dense connective tissue and collagen, which further supports the epidermis [19]. The reticular region contains hair follicles, sweat glands, sebaceous glands, apocrine glands, lymphatic vessels, and blood vessels.

Deeper into human skin, there are numerous glands that play a part in skin lubrication and friction. These glands are commonly referred to as exocrine glands, which secrete substances to an epithelial surface through a duct [20]. There are two main types of exocrine glands: sebaceous glands and sweat glands. Sebaceous glands produce various substances that relate to the skin’s barrier and thermoregulation functions. One of these substances is sebum. Sebum is a multifunctional, oily fluid secreted around the body in various parts. This substance lubricates the hair and skin of mammals and protects the body against outside pathogens [21]. In colder conditions, sebum becomes more lipid, thus repelling water, rain, and snow, keeping the body warm [22]. Sebaceous glands also secrete acids that form an acid mantle with an average pH level between 4.5 and 6.2, which helps repel alkaline elements in contaminating microbes [23]. Another type of sebaceous gland is the areolar gland. Areolar glands are found in the areola surrounding the nipple. 

Sweat glands are the other primary type of exocrine glands. There are two types of sweat glands. There are the eccrine glands, which are the body’s major sweat glands. Eccrine glands are mainly responsible for thermoregulation by cooling skin from water evaporation of sweat [24,25]. Apocrine glands are the body’s other primary sweat glands. These glands are found in specific areas of the body, including the armpit, areola, perineum, ear, eyelids, perianal region, and some parts of the external genitalia [25]. The main purpose of apocrine glands is to secrete sweat thicker than eccrine glands to lubricate hair follicles [26]. Apocrine sweat glands also secrete in periodic spurts, unlike the constant excretion from eccrine sweat glands [27]. Additionally, apocrine glands do not develop in the body until one has reached puberty [28].

Since both glands secrete sweat, it is important to denote what exactly sweat is, the properties that make up a sweat, and the differences in secretion between the glands. Sweat is produced throughout the entire body and is primarily composed of water yet contains trace amounts of minerals, solutes, and other bodily components. In an experiment by Montain, Cheuvront, and Lukaski [29], seven participants completed five, 60-min treadmill exercises. They concluded that sweat is additionally composed of sodium, potassium, calcium, zinc, and magnesium. 

The deepest layer of skin is the hypodermis. The hypodermis serves as a boundary of skin between the dermis and the connective tissue, muscle, and bone below. This layer stores fats and lipids for insulation and cushions the underlying structures. This review article is focused on the upper epidermis and dermis layers as these layers affect skin friction the most. 

## 3. Skin Friction Issues

Human skin comes into contact with various surfaces, textiles, and materials on a daily basis. The friction between skin and clothing textile is an almost unavoidable occurrence that can cause mild to severe issues in certain scenarios. Friction burns, commonly referred to as chafing, are a type of skin irritation caused by friction. The heat generated by friction begins to wear away at the skin’s surface, leading to discomfort. In one example, a person sitting in an office may experience light chafing if their pants or undergarments slightly adjust and crease between their thighs. Ordinarily, this person would likely adjust their seating position or clothing to avoid this mild discomfort. A scenario like this is considered to be a mild inconvenience since the issue can be easily remedied. However, some situations can be much more severe.

In athletic conditions, skin is more viable to shear against clothing from excessive movement. Athletes, primarily those who run, cycle, or wear close-fitting clothing articles and perform rapid movements over an extended period, are more likely to experience skin-clothing friction [30]. During an extended period of exercise, the body thermoregulates itself from internal and external heat. As the body moves and thermoregulates itself, the salt formed from evaporated sweat is abrasive to the surface of clothing articles. Depending on the type of clothing material, weather conditions, body temperature, duration of exercise, and other factors, this experience may be mildly uncomfortable. However, if the exercise increases in duration or intensity, this can lead to clothing removing dermis layers and even result in bleeding. For example, nipple bleeding in the athlete due to excessive friction between skin and cloth. Data from three different studies have shown that 6.7% of joggers in a marathon experience “jogger’s nipple” which is a terminology used for bleeding nipples due to excessive friction. This occurs when the shirt worn by the jogger is made from a coarse fiber such as cotton [30].

Blisters are another issue that can occur from skin–fabric friction. Blisters are small pockets of bodily fluids lying within the epidermis [31]. Friction blisters are primarily caused by a combination of both friction and pressure. These types of blisters are more likely to form on the palms of hands and soles feet where the body experiences intensive loading. There are various exercises and scenarios that can result in friction blisters. For the hands, friction blisters can be caused by gripping and lifting heavy equipment such as dumbbells or barbells. During long walks, runs, hikes, etc., the pressure and strain of withholding and propelling the body forward along with the friction of sock or footwear material can cause blisters of the feet. These blisters commonly cause slight pain and discomfort, but they can lead to disability in certain circumstances [32].

While friction blisters may lead to further issues, they are downplayed because they are commonly seen throughout various fields. Athletes frequently experience friction blisters from vigorous exercise, training, and competitions. The main reason athletes experience blisters is due to the magnitude of the frictional force applied and the number of times the force is applied with the same intensity [30]. Blisters are formed due to horizontal shearing forces that result in splits in the mid to lower Malpighian layer of the epidermis. These separated layers are then filled with blood or tissue transudate [33]. Athletes may experience a decline in performance from the pain. A survey performed at the 28th Combat Support Hospital concluded that 33% of the 872 surveyed enlistees experienced foot friction blisters during their enlistment. Only 11% of the surveyed enlistees who experienced foot blisters sought medical attention and aid for these blisters [34]. Another study was conducted where basic military training recruits were studied to see how blisters are related to other injuries. During this trial of 173 recruits, about 57% of the recruits had developed blisters while performing training activities. It was also later discovered that 53% of the 173 recruits had also developed other injuries in their bodies that were related to overuse or overcompensation. This study concluded that foot blisters are an important factor in the development of overuse injuries in the knees or other parts of the body [35]. The two studies show a big difference in the number of personnel that experienced blisters, 33% in the first and 57% in the second. This is because the first study was voluntary and personnel were not required to take it; however, in the second study, all personnel were required to maintain a diary of blisters experienced [34,35]. If left unchecked, these blisters could cause pain, impairment of focus in active duty, and infection. If pain persists, it could lead to a decline in physical performance and mental fortitude. In military engagement scenarios, friction blisters can be deadly due to the pain of running, walking, or standing, which can possibly inhibit decision making in dangerous situations.

Treating friction blisters is another topic itself. Dressing the blister with an adhesive tape or using a wide area fixation dressing are both recommended [36]. General knowledge suggests keeping the blister clean and limiting the load on the blister. Blisters naturally heal over time; however, Brennan suggests that the best way to treat friction blisters is to prevent them [34].

Pressure ulcers commonly referred to as bedsores are a more severe issue that occurs due to skin friction on clothing and other textiles. The National Pressure Injury Advisor Panel (NPIAP) labels pressure injuries as “localized damage to the skin and underlying soft tissue usually over a bony prominence or related to medical or other devices”. The NPIAP also states that pressure injury “occurs as a result of intense and/or prolonged pressure or pressure in combination with shear”. Pressure ulcers are commonly seen in the elderly, paraplegic, and those bedridden in hospice care facilities and hospitals. As a bedridden person remains at rest, the tissue underlying bones are compressed by the weight of the body against the person’s supporting surface and clothing. This compression results in an obstruction of blood flow, thus limiting nutrient flow to areas of the body. Bedsores are primarily located in regions of the spine, heels, hips, tailbone, shoulders, and back of the cranium [37].

Additionally, friction can further exacerbate pressure ulcers. In hospitals, long term patients are provided attire and beds. Occasionally, it may be required for these patients to be moved elsewhere for treatment or shifted to prevent ulcers from forming [38]. However, if a patient is pulled across the bed the shifting of skin may pull on blood vessels. Additionally, hospital attire may stick to the surface of a patient’s skin and removing said article could result in skin shearing.

The NPIAP states that pressure ulcers come in four primary stages. These conditions from the lowest to highest stages escalate from minor swelling, irritation, and skin hardening to skin and tissue loss. Pressure ulcers can lead to further complications such as anemia, malignant transformations, and sepsis [39]. As a result, pressure ulcers account for a high number of mortality rates in hospitals, with the elderly and paraplegic being the majority affected [37]. Therefore, as we attempt to find tribological solutions for these issues, we must first look at factors that affect these skin friction issues. 

## 4. Skin Friction Factors

The application of tribology can optimize skin friction to either reduce or increase depending on the relevant situation or motion. Coefficient of friction (COF) is defined as the ratio between frictional force and normal load applied. The average COF of different parts of the body against different counter materials is listed in Table 1. It can be seen that different body parts responded differently. For example, the Dorsal forearm yielded lower COF than the Volar forearm, as listed in Table 1. In addition to different body parts, skin friction coefficient depends on various parameters such as age, humidity, skin moisture, materials, anatomical regions, and gender. By studying these parameters, we can reduce the probability of unwanted scenarios, such as chafing or slipping. For example, when we walk into a bathroom with a wet floor, we might want a non-slip film to keep us from slipping. Another example regards humans with a medical condition who develop bedsores when they spend a long time in a particular position. With the use of tribology, we can further reduce unwanted friction-related scenarios to enhance everyday life.

According to Temel et al. [43], human skin is characterized as a viscoelastic material with nonlinear behavior. In recent studies, outside variables such as moisture, the presence of water, and operating conditions are significant factors in skin friction. In relation, Lodén et al. [44] found that skin friction of dry skin was much lower than that of wet or hydrated skin. The skin friction coefficient changes as cosmetic products and lubricants are applied. Depositing ingredients on the skin can lead to noticeable changes in skin adhesion and friction properties. An experiment done by Schwartz et al. [45] observed an increase in skin friction due to sweat, urine, and saline when tested against various materials used in a medical setting. When water evaporates, a thin textured layer accumulates among the skin that consists of salt. This salt layer has a higher coefficient of skin friction than that of other liquids. An increase in the COF is directly related to an increase in adhesion bonds that develop between the skin and the material.

**Figure 3:**
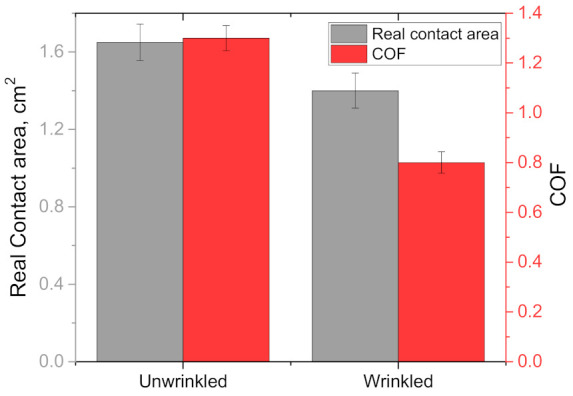
The effect of wrinkles on the real contact area and COF against dry PET flat surface at 2 N normal load.

A study conducted by Lin et al. [46] showed that when then the skin is fully saturated in a lubricant, the skin produces water-induced wrinkles. The wrinkles increase the apparent contact area and decrease the real contact area. These results change the real/apparent contact area decreased for wrinkled finger as shown in Figure 3. The contact area in this study was measured by the ink method using contact pads. The data for Figure 3 was extracted from the study conducted by Lin et al. [46]. Furthermore, the effect of lower real contact area by the presence of wrinkle reduced the COF in all three test conditions, i.e., dry finger to dry surface (shown in Figure 3), dry finger to wet surface, and submerged water condition.

Similar to this study, Kareklas et al. [47] experimented with glabrous skin on human hands and feet that is controlled by the autonomic nervous system. The experiment was designed to test if water-induced wrinkles improved the handling of objects underwater. The experiment concluded the wrinkles did improve the handling of objects in a saturated environment. The skin experiences an increase in friction when exposed to a lubricant for a period of time (before wrinkle forms), then decreases when water-induced wrinkles occur [3]. Therefore, without any wrinkles, dry skin has a lower COF compared to fully saturated skin or wet skin.

### 4.1. Anatomical Regions 

Different regions of the body have varying skin friction coefficients. Regions, such as the finger pad, palm, ventral forearm, and elbow will react differently to a friction test. Each of these anatomical regions serves a different purpose for interaction with materials. Vilhena et al. [48] evaluated the friction on the finger pad, palm, ventral forearm, and elbow regions with different lubricating conditions. The lubrication conditions were natural skin, wet skin, and lubricated skin with Vaseline against a textile. 

The COF was measured by using a portable measuring probe. The probe was equipped with different fabrics that would be found in a hospital. The fabrics are bed linen, foam dressing, an adult diaper, and a bed protector. The probe is also equipped with a multi-component force sensor that measures the normal and tangential forces. The COF can be measured by measuring the tangential forces. 

**Figure 4:**
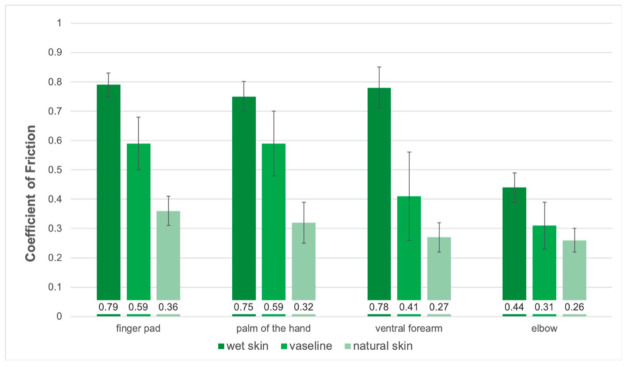
Measured skin friction coefficient from a portable measuring probe equipped with a reference hospital fabric under different lubricating conditions, Reproduced from [48].

The results from the experiment are shown in Figure 4, which was redrawn using data from the paper, revealing that wet skin had the highest skin friction coefficient while natural skin showed the least skin friction coefficients for different regions of the arm. The finger pad, palm, and ventral forearm had a COF of 0.77, while the elbow had a COF of 0.44 for wet skin. The COF for the finger pad and palm was 0.59 when the skin was lubricated with Vaseline. For natural skin conditions, the COF of the ventral forearm and elbow was 0.26, and the finger pad had a COF of 0.36.

The data from Vilhena et al. [48] is different than that of Sivamani et al. [3]. Sivamani et al. [3] found that the COF of wet skin experienced less friction than that of lubricated skin due to the changes in the real/apparent ratio. The discrepancies of this data can come from the amount of time the skin was saturated in a liquid. Vilhena et al. [48] applied a soaked napkin for 5 min to the skin, whereas Sivamani did not list the time, and it is expected that it is long enough to form wrinkles that decreased the real apparent area ratio and led to higher COF. 

### 4.2. Humidity

Humidity is the measurement of water in a particular environment and changes with temperature. A study conducted by Schwartz et al. [45] looked at the effects of humidity on skin friction against medical textiles. Schwartz’s study focused on the friction of porcine skin and textiles that would be found in a medical environment with various moisture conditions. The study obtained three medical textiles friction coefficients against the skin. The test used adult diapers, cotton, and polyurethane dressing to determine the COF. The test used a tilting-table electric tribometer to quantify the friction coefficient against the medical textiles. The experiment started with dry skin then transitioned into the presence of sweat, urine, and saline. Sweat, urine, and saline were applied to the textile samples with $8\;\mathrm{mg}/{\mathrm{cm}}^{2}\;$ which is the 24 h mean transepidermal water loss quantity. It was found that the COF of dry skin against the adult diaper and polyurethane was 0.59 and 0.91, respectively. The soaking of diaper and polyurethane in sweat led to a higher COF of 0.85 and 1.19, respectively. The results of these experiments demonstrate that the porcine skin COF against the textiles increased with the addition of sweat, urine, and saline. The COF increased the most with sweat or saline, however, it did not increase as much with urine. The highest COF was observed in the polyurethane material which shows that material type also influences skin friction.

### 4.3. Counter Materials and Fabric Type

**Figure 5:**
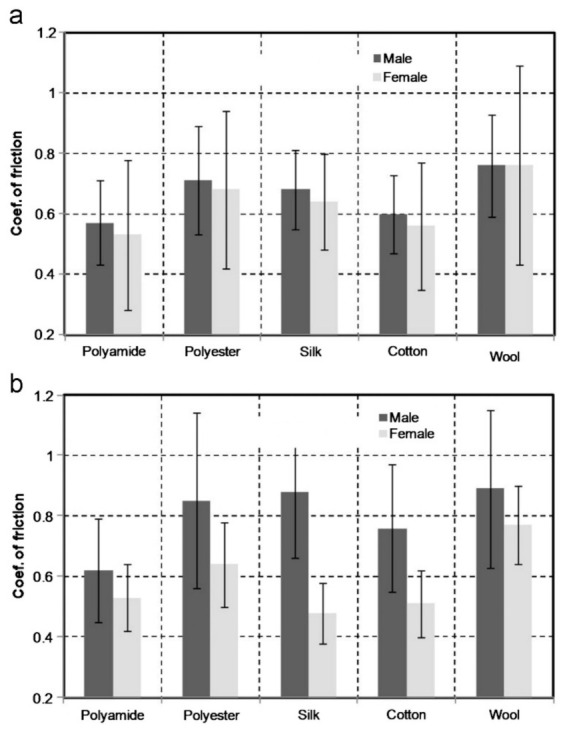
COF against the skin for various fabrics: (**a**) forearm and (**b**) palm [50]. Reproduced with permission from Ramalho, A.; Szekeres, P.; Fernandes, E., Friction and tactile perception of textile fabrics; published by Elsevier, 2012.

Counter textile materials are another factor that greatly affect textile-skin friction. In Table 1, there are different anatomical regions as well as different counter materials that show that friction coefficient changes with the type of material. Cotton is a common fabric used in clothing. Different loads and velocity can greatly influence the friction between cotton and other surfaces. Chen et al. [49] investigated the influence of load and velocity on the tribological properties of cotton fabric. The study showed that as velocity increased, the friction decreased initially and then increased due to the elastic deformation of the cotton fiber. A correlation between tactile perception and friction coefficient can be made where rougher, coarser, and more adhesive fabrics show a higher friction coefficient when rubbing against skin [43,50,51]. A study conducted by Ramalho et al. [50] assessed the friction values of human skin sliding against different fabrics. The tests were conducted using a handheld probe that was rubbed against two different anatomical regions: the palm and ventral forearm face. The five different types of fabrics used for the test were polyamide, polyester, silk, cotton, and wool. The COF results of the test are shown in Figure 5 for two regions of the body. The results show that wool had the highest COF for both anatomical regions, whereas polyamide-based fabric showed the least COF in all cases except for the palm of a woman. The textiles that showed lower COF values, polyamide, and cotton, are more hydrophilic. The study also recorded the sensation experienced by the subjects upon touching the different textiles. Wool and silk were rougher and more adhesive to the touch, whereas polyamide and cotton were smoother and more slippery [50]. 

## 5. Skin Friction Controlling Factors

Keeping the skin “comfortable” is very important. It has been made clear that various problems may develop with the skin at the skin–textile material interface in the form of blistering, chafing, and various other skin irritations. Not only can the skin itself be affected but, injury to the skin may lead to other more serious health issues such as infection or even overuse and overcompensation injuries, leading to damage to joints, ligaments, and muscles. For example, in a study of a 21-day march of volunteer college students, 95% of the 142 volunteers had some sort of foot-related injury. Of these march-related injuries, excluding foot injuries, 12% were ankle pain, 13% were knee pain related, and 8% of the injuries had to do with Achilles tendon pain. Of the volunteers, about 4%, or six volunteers, who developed blisters or other foot injuries repeatedly needed extra medical attention for a two-week period to recover and return to their normal daily lives. The participants that did not develop foot injuries did not require extra medical attention or time to return to their normal lives [52]. Another study by Knapik et al. [53] showed that 4% of the 180 soldiers who marched had to discontinue due to blister formation and another 4% needed additional time after the march to fully recover. Something must be done to combat this skin–textile material friction problem experienced by almost every individual worldwide, thus, what are the options?

One way to reduce friction at the skin–textile material interface is the introduction of lubrication. Just like a tribological application of oil between two metallic mating pairs, there are lubricants made to be applied to either the skin or the material in areas where rubbing occurs in order to reduce friction. Common options available to the public are “off the shelf” creams and gels made to reduce friction and provide protection from blisters or other friction-related injuries on the skin, such as BodyGlide, Hydropel, and Nok, among various others [54]. The function of these creams and gels is to reduce the friction between skin and textiles. These creams operate in three phases during the application process. When the cream is first applied, it is thick and operates in the hydrodynamic or mixed lubrication regime which is the first phase. The skin feels slippery and COF between skin and the cream in this phase is low. The cream is then absorbed by the skin and operates in the boundary phase. This is the second phase where the skin feels sticky, and the COF and adhesive force is high. In the third phase, due to some physical and chemical changes on the surface of the skin, the COF and adhesive force is low [55]. Typically, this is where the difference between high-quality creams and low-quality creams lies. High-quality creams can retain the high friction properties between skin and cream which allows the cream to stick to the skin for longer and provide better lubrication. 

Another effective measure at preventing skin injury is simply keeping the feet or other problem areas dry or by using suitable lubricants based on skin type, as discussed in previous sections of this paper. This comes in the form of wicking moisture away from the skin. Moisture can be wicked away in various ways, such as the application of skin protectants [56]. Adding moisture-absorbing powders such as cornstarch or talcum powder will allow for the sweat from the skin to be drawn away from the skin–textile material interface. Similarly, certain textiles themselves are very absorbent and will allow for moisture to move away from the surface of the skin. A specially developed material containing silica aerogel within its fabric weave can provide its wearers with high breathability as well as permeability. This unique fabric can easily transfer water vapor through its structure to the atmosphere and vice versa [57]. Another type of moisture wicking fabric was studied that used a hydrophobic and hydrophilic layer to effectively transport moisture along with the fabric. The constructed material was an electro-spun dual layer nanofibrous mat that consisted of an outer and inner shell. The outer shell was made from base treated Cellulose Acetate (CA) and the core shell was made from a polyacrylonitrile (PAN) rich core and poly(vinylidene fluoride) (PVDF) shell [8,58]. In other words, both fabrics are highly breathable and allow for effective transfer of moisture away from the body, which in turn helps to reduce friction. 

Friction at the skin–textile material interface may also be reduced by using different materials that may lower the COF at the interface, reducing the magnitude of overall frictional forces and shearing forces within the skin that cause injury. An effective way to reduce the COF is with an “off the shelf”, friction-reducing shoe patch, which provides an exceptionally low friction level, lasts 300 miles, and allows your sock to protect your skin [54]. This reduces the friction at the sock-shoe interface by applying low friction patches to problem areas; however, this would not work well in other areas of the body where there is the interaction of skin–textile material. Another product that can reduce friction against the skin in a low impact way is called PreHeel+. This spray-on barrier provides a low friction layer that can last up to 6 h in normal use [59]. This barrier provides low friction at any place it is applied. It has the drawback of becoming ineffective if exposed to a high wear situation or if too much moisture is present. It is still a decent alternative for the average person for occasional use. Perhaps a very important application of reducing friction between human skin and textile material is the use of low friction bed sheets in hospitals. Low friction sheets such as EZ Move Slide Sheets help in the movement of bedridden patients when it comes time to turn the patient or change the bed sheets. This is achieved by ultra-low friction material, which makes it safer and more comfortable for patients as they are being moved [60]. It is common for bedridden patients to develop pressure ulcers or bed sores due to shearing forces of skin against the bedding material [61].

Clothing textile materials themselves may be changed or optimized in order to reduce the COF and wick or absorb moisture away from the skin. This option will be the simplest choice in friction reduction at the skin–textile material interface for users because it creates an option that allows people to forget about having to apply products or alter their existing clothes or equipment. Some sports and activities, such as lifting, implement the use of chalk to absorb moisture in the hands and maximize performance [62]. According to Ishikawa [63], “Textiles can be engineered to ease the slippage of fabrics on the human skin by both the fiber composition and construction of fabrics”.

## 6. Textile Design and Testing 

After reviewing the anatomy of human skin, friction factors that have an effect on the skin, and the importance of reducing friction on the skin’s surface, it has been determined that one of the best ways to reduce friction at the skin–textile material interface is through surface engineering of the materials that interact directly with the skin. This allows for the root cause of friction on the skin to be addressed rather than trying to focus on solutions that may only provide temporary relief from skin irritation. This section focuses on how and what is being done to investigate how textile materials interact with human skin to study and understand friction at the skin–textile material interface. An important factor of the study of skin–textile material interaction is how to accurately and consistently measure the friction of different materials against human skin. In a study involving the foot and sock friction, two different methodologies were used in order to obtain data about friction between socks and human skin. 

The first method used to test material friction against the skin was a custom-built rig that consisted of a friction plate, sock material, and load cells. These load cells were capable of measuring both normal and shear forces present during sliding at the skin–textile material interface. This method required volunteers to slide their own feet across the testing plate with the load cells. They were to maintain a speed as steady as possible [64]. This method may introduce many human-based errors. This is why the second friction testing method utilized a mechanically driven loading device. Volunteers simply had to stand on a platform where a probe was applied to the plantar section of the foot. The probe is then able to measure shear and normal loads [64]. At the end of the experiments, it was concluded that both methods of measurement on foot allow friction values to be calculated from the data collected from the measurement rigs. Both friction measurement devices produced data that was in agreement, so both methods have been determined as effective measurement methods for foot friction. 

Other ways to accurately measure material friction consist of the use of an LPMT (Laboratoire dePhysique et Mécanique Textiles). The device was developed to obtain measurements of friction forces of textiles in moving contact with simulated skin material. The special skin-like material used in the apparatus is a material called LoricaSoft. This material provides a surface structure that can realistically simulate human skin in dry conditions [65]. The use of this material coupled with a mechanical test apparatus allows for human error in experiments to be removed in order to focus on studying the materials alone.

**Figure 6:**
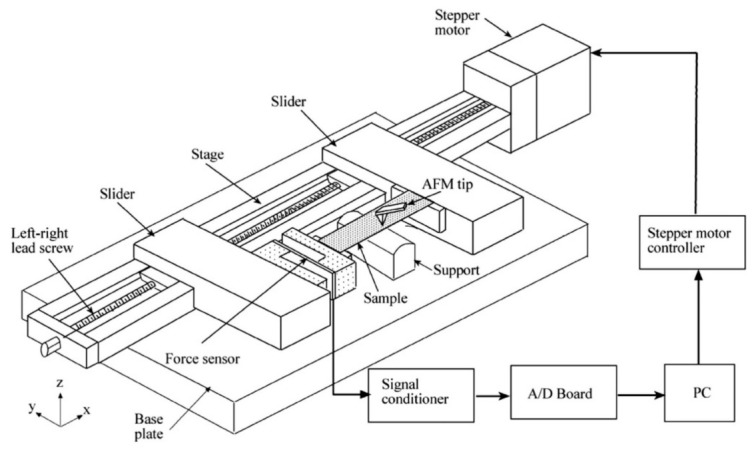
Custom-built setup for testing tribological properties of skin at a nano level [55]. Reproduced with permission from Bhushan, B., Nanotribological and nanomechanical properties of skin with and without cream treatment using atomic force microscopy and nanoindentation; published by Elsevier, 2011.

**Figure 7:**
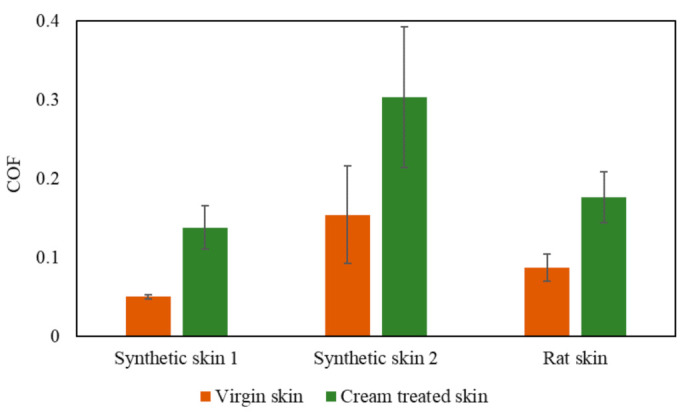
COF values for nanoscale tested synthetic skins and rat skin with and without cream.

Tribological properties of skin have also been studied on a nanoscale as most surfaces are not smooth and interactions between skin and other surfaces occur at a microscopic level. Understanding the effects on a nanoscale can help to obtain a better understanding at a macroscale. To study such interactions at a nanoscale, studies done by Bhushan et al. have used atomic force microscopes (AFM) and nano indenters to measure the tribological effects of skin with and without cream [55,66,67]. A custom-built stage was made which was attached to the base of the AFM, which was in turn connected to a stepper motor that applies a tensile load on the skin sample as shown in Figure 6. Tribological studies were done to compare two synthetic skins and rat skin with and without cream using AFM and nano indenters. The COF results were extracted from the study and presented in Figure 7. The results showed that COF increased after the application of cream that resulted from the presence of a cream film. The viscous friction of the tip with the cream film resulted in an increase in the overall frictional force as the tip slid across the skin–cream film [66]. 

**Figure 8:**
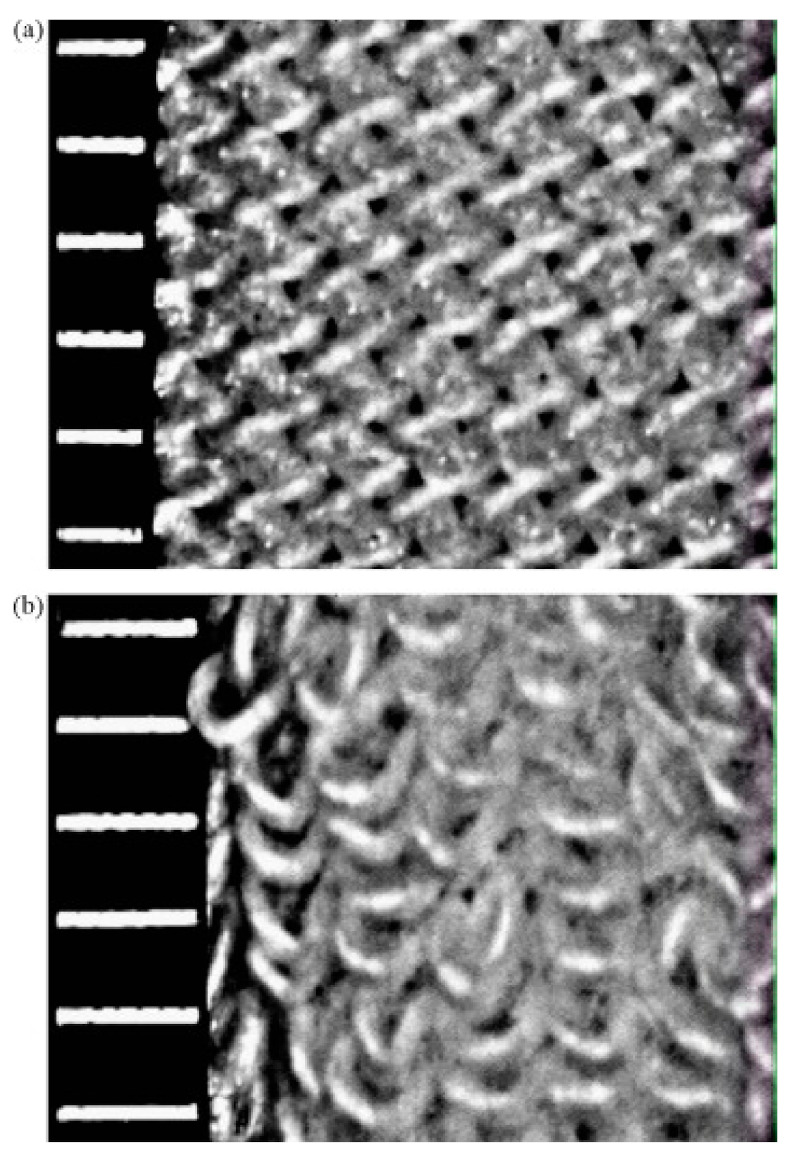
Sock structure: (**a**) basic terry which has a single cotton yarn structure and (**b**) jersey terry which has a second yarn to form stitches on the back yarn [68]. Reproduced with permission from Baussan, E.; Bueno, M.A.; Rossi, R.M.; Derler, S., Experiments and modelling of skin-knitted fabric friction; published by Elsevier, 2010.

**Figure 9:**
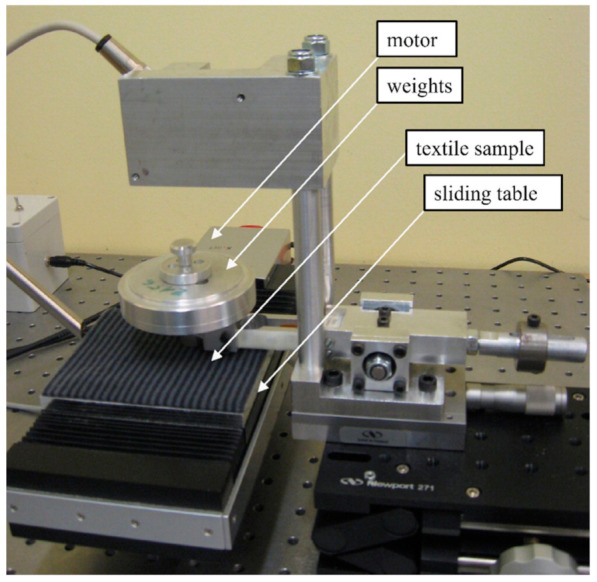
Custom-built reciprocating linear tribometer used to simulate friction between foot and sock [68]. Reproduced with permission from Baussan, E.; Bueno, M.A.; Rossi, R.M.; Derler, S., Experiments and modelling of skin-knitted fabric friction; published by Elsevier, 2010.

A study conducted by Baussan [68], investigated how sock structure influenced friction for the recreational runner. Two types of cotton sport socks—basic jersey and terry jersey—with different knitted structures were tested, as seen in Figure 8. The pressure exerted on the foot arch by the shoe–sock system was examined in runners. The friction contacts between foot and skin were simulated using a reciprocating linear tribometer which was developed for this experiment (Figure 9). The results of the simulated test showed that the friction force of the basic terry was ~1.35 times lesser than that of the jersey terry due to the hairiness of the jersey terry structure.

In a similar study, Keiko Suganuma [69] investigated the different factors that affect fabric “slipperiness”. This study determined that there are three most important factors of fabric slipperiness: the fuzziness of fabrics; the linearity of the weave texture, and the real contact area. In this study, 62 different commercial fabrics were investigated in order to understand fabric slipperiness and its effects on friction, not only on human skin but also related to other factors, such as safety aspects involved when textiles are used—i.e., moving patients in a medical setting—and, even how these factors could play a role in the process of washing clothes in a washer machine. Another study by Hosseinali [70], investigated the frictional properties of different varieties of cotton with different fiber assemblies. They showed that there was a positive correlation between short fiber content and COF. The simulations done in this study also show that fiber friction depends on surface properties, fiber dimensions and mechanical properties. 

In addition to synthetic polymeric materials, researchers have given increasing attention towards biopolymers for textile fabrication. Various biopolymers have been utilized for textiles such as chitosan, cyclodextrin, sericin, and alginate [71]. These biopolymers are not only eco-friendly in nature but also carry antimicrobial properties. The antimicrobial property of these biopolymeric textiles can prevent harmful effects of microbial pathogens, such as disease transmission, offensive odors, and deterioration of textiles. Moreover, these biopolymers, such as polysaccharide on the polyester (PET) woven fabric, have been studied as drug delivery textile materials while characterizing the tribological comfort [72]. The study revealed that with the addition of biopolymer (polysaccharide) on PET substrate in loaded drug delivery textile, the COF reduced by 7–29% compared to PET substrate with the loaded drug. Therefore, the biopolymers do not only provide drug delivery functionality but also support skin care by reducing friction. However, more tribological attention is required to develop biopolymeric textile materials, since the tribological studies against in vivo or in vitro skin are limited on this material. 

The other trending technology in the field of the textile and fashion industry is 3D printing [73]. The 3D printing technology allows for the usage of recycled polymers, as well as provides the capacity to fabricate intricate shapes. Similar to biopolymer textiles, the tribological studies on the 3D printed textiles or accessories are limited against the skin. For example, Kasar et al. [74] studied the 3D printed thermoplastic polyamide (TPA) and thermoplastic polyurethane (TPU) material against the skin model (artificial skin prepared using gelatin and cotton). The COF of TPU and TPA increased in wet conditions compared to dry conditions as the skin model replicated wetting characteristics of the skin. To provide lower friction even in the presence of simulated sweating conditions (wet condition), the authors designed the 3D printed layers with 80% fill density and captured sodium polyacrylate crystals between the 3D-printed layers. The porosity in the printed TPA and TPU allowed the water to pass through and be absorbed by the sodium polyacrylate crystals. Thus, the skin model did not soften and provided lower COF. This study demonstrates that 3D printing technology can be employed in the textile industry while providing tribological comfort. 

## 7. Summary and Future Outlook

Friction is an important factor in everyday life. There is no doubt that making the skin comfortable is an important topic that can benefit people all over the world, from athletes and remote area hikers, hospital patients, to even the average person going about their daily lives. Skin comfort is often a neglected subject until it becomes a problem that cannot be avoided and must be dealt with. If left unattended, simple injuries from skin–textile material interaction may lead to painful injuries that can take weeks to heal. These injuries may manifest into more extreme cases creating portals for infection to enter the body and cause illness. It has even been proven that obtaining injuries on the skin surface, which is very common, may lead to overuse injuries elsewhere in the body—potentially creating long-term effects on one’s overall physical health. Therefore, it is important to improve the understanding of tribological interaction between clothing/fabrics and human skin. A better understanding of this topic can not only increase the comfort and effectiveness of clothing articles for performance uses but, can also aid in medical uses, among other useful applications. Lubricants cause the highest change in the coefficients of frictions. Generally, it has been observed that dry skin experienced the lowest COF in the absence of skin wrinkles, and once wrinkles are present, the COF decreased even in wet conditions due to lesser real area of contact against different textiles. 

It has been proven that there are many different ways to make the skin more comfortable, from topical treatments to engineering materials to better suit the skin–textile material interface. The usage of multifunctional biopolymers and 3D printing technology has also been highlighted for textile applications. There have been many methods developed to test skin–textile material friction which have helped design new textiles for a wide variety of applications. One of the methods described is the LMPT device, which is used to measure frictional forces of textiles in motion against skin-like material or artificial skins. Another device mentioned was custom-built, using a probe to measure shear and normal loads on the foot. The two forces measured were then used to calculate the COF. These testing devices can aid in the design of new materials that provide comfort for many different activities.

Skin–textile material friction can be greatly improved by designing materials in such a way that they are comfortable and provide low friction. In this regard, there have not been many helpful simulation studies conducted in this field. Various patterns and textile models can be built using simulations which can make it easier to test many skin–textile material friction conditions. Tactile perception of textile is another area that has not been extensively studied. It is important to understand tactile perception as it correlates to friction. The use of 3D printing could be used to produce the materials and the skin–textile material friction could be further studied. The difference in COF between traditionally produced and 3D printed materials could also be compared to see which method produces better materials. These methods can effectively help our understanding of skin–textile material friction and help produce better materials for different applications.

## Figures and Tables

**Table 1:** COF on untreated normal skin of different body parts with respect to different counter materials.

Body Part	Counter Materials	COF	Reference
Dorsal forearm	Glass	0.24	Koudine et al. [40]
Volar forearm		0.64	
Forehead	Teflon	0.34	Cua et al. [1]
Volar forearm		0.26	
Palm		0.21	
Abdomen		0.12	
Upper back		0.25	
Forearm	Teflon	0.48	Elsner et al. [41]
Vulva		0.66	
Inner forearm	100 Cr 6	0.5	Kwiatkowska et al. [42]

## Data Availability

This study did not report any datasets analyzed or generated.
